# Malaria care-seeking behaviours and infection prevalence among short-term Myanmar migrants in Thailand

**DOI:** 10.1186/s12936-025-05539-8

**Published:** 2025-09-01

**Authors:** Pyae Linn Aung, Piyarat Sripoorote, Nichakan Inthitanon, Yupaporn Wattanagoon, Raph L. Hamers, Jennifer Ilo Van Nuil, Liwang Cui, Wang Nguitragool, Leigh Jones, Anindita Gabriella Sudewo, Jetsumon Sattabongkot, Daniel M. Parker

**Affiliations:** 1 Mahidol Vivax Research Unit, Faculty of Tropical Medicine, Mahidol University, Bangkok, Thailand; 2 Department of Clinical Tropical Medicine, Faculty of Tropical Medicine, Mahidol University, Bangkok, Thailand; 3 Centre for Tropical Medicine and Global Health, Nuffield Department of Medicine, University of Oxford, Oxford, UK; 4 Oxford University Clinical Research Unit Indonesia, Faculty of Medicine, University of Indonesia, Jakarta, Indonesia; 5 Oxford University Clinical Research Unit, Ho Chi Minh City, Vietnam; 6 Division of Infectious Diseases and International Medicine, Department of Internal Medicine, Morsani College of Medicine, University of South Florida, 3720 Spectrum Boulevard, Suite 304, Tampa, FL 33612 USA; 7 Department of Molecular Tropical Medicine and Genetics, Faculty of Tropical Medicine, Mahidol University, Bangkok, Thailand; 8 Department of Population Health and Disease Prevention, Department of Epidemiology & Biostatistics, University of California, Irvine, CA USA

**Keywords:** Malaria, Healthcare seeking, Prevalence, Migrants, Myanmar, Thailand

## Abstract

**Background:**

The recent resurgence of malaria in western Thailand has coincided with increased cross-border migration from Myanmar following political unrest. As short-term migrants from endemic areas may contribute to sustained local transmission, this study examined their malaria care-seeking behaviours and infection prevalence.

**Methods:**

A community-based cross-sectional study was conducted during March–April 2025 in six malaria-endemic villages of Tha Song Yang District, Tak Province, western Thailand. A structured questionnaire was administered, including a nine-item section on care-seeking behaviours. Malaria prevalence was determined by PCR testing of dried blood spot samples. Determinants of care-seeking scores were analysed using a generalized linear model, and infection risk was estimated using Firth logistic regression.

**Results:**

Among 300 participants (mean age: 34.5 ± 14.5 years; 47.3% male), over 60% recognised the need to seek care for fever or chills, yet only 35% reported doing so within 24 h of symptom onset. Although 98.0% preferred public health facilities, only 50.3% had ever visited one for suspected malaria. Higher care-seeking scores were associated with being a daily wage labourer (β = 0.66; 95% CI 0.01–1.31), infrequent return to Myanmar (β = 1.34; 95% CI 0.05–2.62), prior malaria experience (β = 1.08; 95% CI 0.59–1.58), and higher malaria knowledge (β = 0.34; 95% CI 0.24–0.44). Karen ethnicity was negatively associated with care-seeking (β = − 0.95; 95% CI − 1.74 to − 0.16). Six (2%) afebrile *Plasmodium vivax* infections were detected. Lower malaria knowledge (OR = 13.5; 95% CI 1.58–177.0) and care-seeking scores (OR = 5.86; 95% CI 1.15–57.7) were significantly associated with infection.

**Conclusions:**

Despite generally positive attitudes toward malaria, self-reported timely care-seeking among short-term Myanmar migrants remained limited. Socioeconomic status, migration patterns, ethnicity, and malaria knowledge significantly influenced care-seeking behaviours. The detection of asymptomatic *P. vivax* underscores the need for migrant-focused surveillance and targeted health education to support malaria elimination efforts.

**Supplementary Information:**

The online version contains supplementary material available at 10.1186/s12936-025-05539-8.

## Background

Malaria remains a significant global health challenge, with the highest burden reported in the World Health Organization (WHO) African Region, followed by the WHO South-East Asia (SEA) Region [1]. Achieving malaria elimination in the SEA Region by 2030 appears unlikely due to several persistent challenges, including the emergence of artemisinin resistance and ongoing transmission in border areas [1–4]. Border malaria is a major obstacle to elimination, as neighbouring countries often share geographic and environmental conditions conducive to malaria vector proliferation. Many border areas also experience substantial cross-border migration, further elevating the risk of transmission [4, 5]. Notably, the border regions between Thailand and Myanmar, as well as Thailand and Cambodia, have been associated with multidrug-resistant malaria strains, complicating elimination efforts [6]. Given these challenges, intensified and targeted interventions are essential to address border malaria and support regional elimination goals.

In 2021, Thailand reported 3279 malaria cases, primarily in provinces along international borders. By 2024, this number had increased to approximately 16,000, with over 90 percent of cases occurring in border provinces. Tak Province, located along the Thailand–Myanmar border, accounted for more than half of the national total [1, 7–10]. A large proportion of these cases were among Myanmar migrants, driven in part by population displacement following the political crisis in Myanmar after 2021. The continued transmission in border regions, coupled with the presence of primary malaria vectors including *Anopheles minimus*, *Anopheles maculatus*, *Anopheles annularis*, *Anopheles barbirostris*, and *Anopheles dirus* [11–13], suggests that the influx of migrants could further intensify malaria transmission. These circumstances highlight the need for malaria control measures specifically tailored to migrant populations.

Thailand is home to an estimated 4–5 million migrants, with approximately 80 percent originating from Myanmar. These migrants are commonly classified as either long-term (residing in Thailand for more than 6 months) or short-term (less than 6 months) migrants [14]. Although Thailand provides free malaria diagnosis and treatment to all individuals regardless of legal or migration status, access to these services remains limited [15]. Temporary migrants, in particular, often reside in remote or hard-to-reach areas and face challenges related to high mobility. Social and cultural barriers, including marginalisation, discrimination, and limited integration into host communities, further hinder access to services [16, 17]. A recent study reported that only 27.3% of Myanmar migrants in Thailand had adequate access to malaria-related services, including diagnostics, treatment, prevention tools, and health information [15].

Language barriers often prevent migrants from participating in health education activities such as community talks and printed materials. Undocumented migrants may also avoid public health facilities due to fear of legal consequences [18], while financial concerns and perceived costs can further discourage service utilisation [19]. Preventive behaviours, such as consistent bed net use, remain low, and early care-seeking as well as adherence to treatment regimens, especially the full 14-day primaquine course, are often inadequate [20–23]. These issues contribute to continued localised transmission. In addition, many migrants serve as reservoirs of infection, often due to partial immunity, incomplete treatment, or reliance on unregulated pharmacies and traditional medicine [15, 24–27]. These persistent challenges highlight the limitations of current malaria interventions and reveal critical gaps between programme implementation and the specific health needs of migrant populations. Given the increasing number of malaria cases in Thailand and the limited attention paid to newly arrived migrants, it is essential to understand care-seeking behaviour and infection status in this vulnerable group. This study aimed to examine malaria care-seeking practices and assess the prevalence of malaria among short-term Myanmar migrants in Thailand. Understanding and addressing barriers to timely diagnosis and treatment in this mobile population is essential, as accurate detection and prompt treatment of imported malaria cases are critical to achieving Thailand’s goal of malaria elimination by 2030.

## Methods

### Study design

This study employed a cross-sectional, community-based exploratory design targeting temporary Myanmar migrants residing in Thailand’s border areas. Data collection was conducted in late March and April 2025.

### Study site

**Fig. 1:**
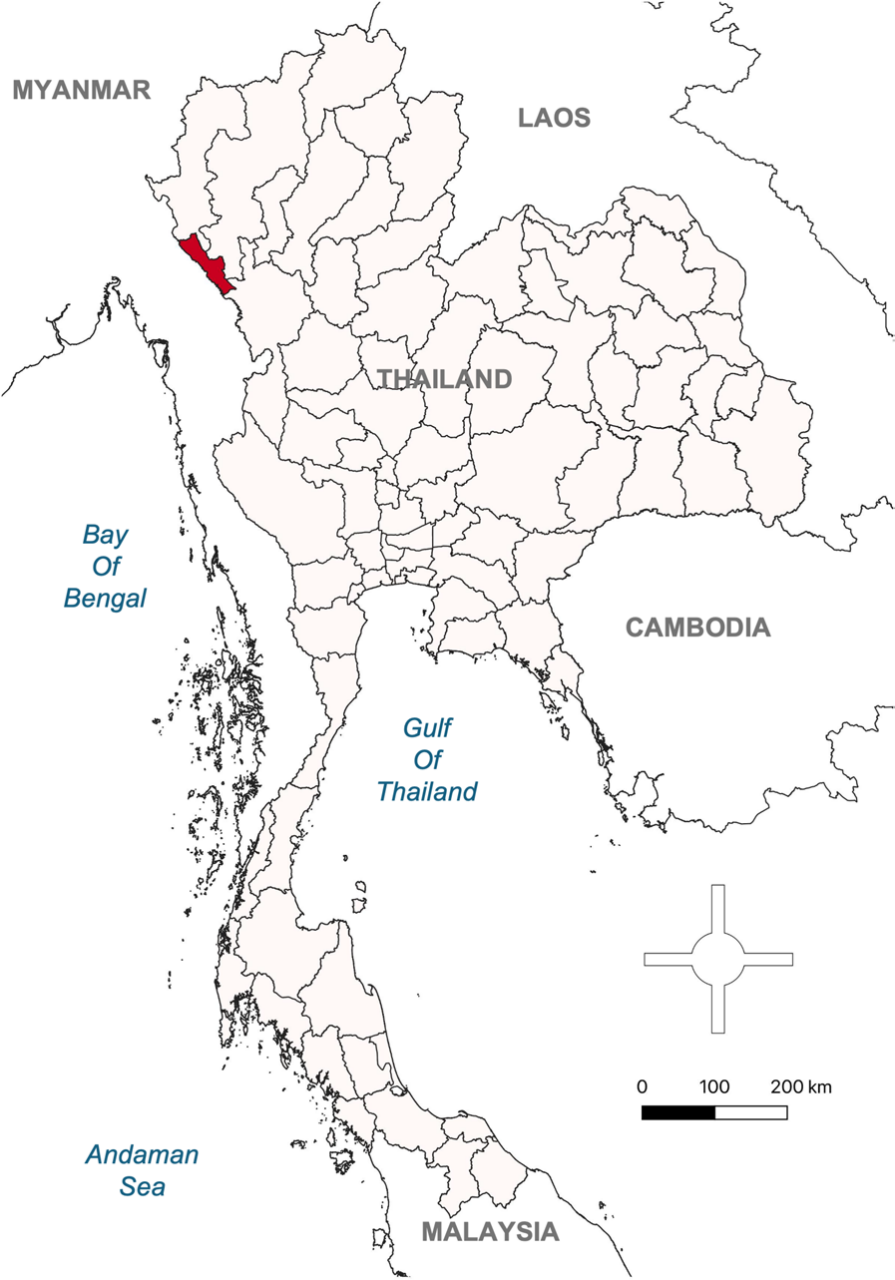
Map showing the study site, Tha Song Yang District in Tak Province (highlighted in red), located along the Thailand–Myanmar border in western Thailand. The base map shapefiles were obtained from the DIVA-GIS database (https://diva-gis.org) and modified using QGIS for Mac (version 3.34.2-Prizren)

The study was conducted in Tak Province, located in western Thailand, a region with persistently high malaria burden. Among its nine districts, Tha Song Yang was purposively selected due to its high malaria prevalence and the significant influx of migrants from Myanmar. The district borders Kayin State in Myanmar and is separated by the Moei River, which is easily crossed on foot during the dry season (Fig. 1). Tha Song Yang has an estimated population of 95,000, approximately 10–20% of whom are Myanmar migrants. In 2024, around 2000 malaria cases were reported in the district, a fivefold increase compared to 2021 [7]. Based on consultations with local malaria stakeholders and community leaders, six villages with the highest malaria prevalence and large migrant populations were selected as study sites.

### Sample size and sampling

The required sample size was calculated using the infinite population formula, [28] with a reference proportion of 21.0% of migrants reportedly not seeking appropriate treatment for malaria-like symptoms, [15] and a 5% margin of error. After accounting for a 10% non-response rate, the final target sample size was 300 participants.

Village leaders in the selected sites provided lists of migrants. Temporary Myanmar migrants, defined as individuals residing in Thailand for less than 6 months, were systematically sampled. An average of 50 participants was recruited from each selected village. Eligible participants included male and female migrants aged 18 years or older, regardless of documentation status. Individuals who had been diagnosed with malaria within the past month were excluded to minimise the risk of underestimating malaria prevalence and potential detection of residual parasite DNA following treatment [29]. However, detailed treatment history beyond the past month (e.g., drug regimen, timing of last dose) was not collected. Migrants who crossed the border frequently or returned daily were eligible. Those who were critically ill or appeared to be under the influence of drugs or alcohol at the time of interview were excluded based on observation.

### Data collection

Two components of data collection were implemented: (1) a structured questionnaire, and (2) dried blood spot (DBS) samples for malaria testing. The questionnaire was adapted from the WHO Malaria Indicator Survey [30] and other validated sources [23, 31–34], and translated into Thai using a back-translation approach. It included items on demographic characteristics, malaria-related knowledge, attitudes and care-seeking practices (Supplementary file 1). Employment type (e.g., daily wage labour, agriculture, others) and border-crossing frequency (daily vs. infrequent) were recorded as separate variables because these groups are not mutually exclusive. For the knowledge and care-seeking sections, sub-questions with predefined response options were included. The knowledge section comprised 11 questions, while care-seeking behaviour was assessed using nine items. Participants could select more than one correct response per item. Each correct answer was awarded one point, and incorrect answers received zero points. The attitudes section included 11 statements (measured on a 4-point Likert scale), [35] incorporating both positively and negatively worded items. Negative statements were reverse coded during score calculation.

Because Myanmar migrants are often beyond the reach of routine surveillance and direct communication could be challenging, the study leveraged the support of local Village Health Volunteers (VHVs). VHVs shared similar socioeconomic and linguistic backgrounds with the migrants, with many fluent in both Thai and Karen—the most commonly spoken dialects among border communities. VHVs with prior experience in malaria-related duties were trained over 1 day, which covered Good Clinical Practice (GCP), the informed consent process, questionnaire content, DBS collection, and verbal back-translation from Thai to Karen. Practical sessions were included to standardise survey and specimen collection procedures.

Trained VHVs then visited the residences of selected migrants to conduct data collection. Each participant was administered a structured questionnaire survey. Filter papers were pre-labelled and distributed by the research team. Two DBS samples were collected via finger prick, air-dried, and stored in sealed envelopes with desiccants. Each structured survey, including DBS collection, lasted approximately 30 min. No blood slides were collected for microscopy because the study used an active case detection approach among apparently healthy individuals, in whom malaria prevalence is typically low. However, participants presenting with fever at the time of the survey were referred to malaria clinics for further management.

All DBS samples were transported to the laboratory, stored at room temperature for less than 1 month, and processed for malaria diagnosis using genus-specific qPCR [36]. Samples that tested positive were further analysed using species-specific qPCR targeting each of the five human malaria species–*Plasmodium falciparum*, *Plasmodium vivax*, *Plasmodium malariae*, *Plasmodium ovale*, and *Plasmodium knowlesi* [37, 38]. Screening was conducted using PCR due to its greater sensitivity compared to commonly used diagnostic methods such as microscopy and rapid diagnostic tests, particularly for detecting low-density infections in asymptomatic individuals. No immediate diagnostic results were provided because PCR requires laboratory processing, and current national guidelines do not permit treatment based solely on PCR-positive results [39]. However, the list of PCR-positive individuals was communicated to the respective malaria clinic staff through established channels for further appropriate follow-up as needed.

### Data analysis

Data were entered into CSV files, and all statistical analyses were performed using RStudio version 2025.05.0 + 496 (R Foundation for Statistical Computing, Vienna, Austria). Descriptive statistics were used to summarise participants’ sociodemographic and behavioural characteristics. Continuous variables were reported as means, standard deviations, and ranges, while categorical variables were summarised as frequencies and percentages. Violin plots with embedded boxplots were used to visualise the distributions of malaria-related knowledge, attitudes, and care-seeking scores.

To identify factors associated with malaria care-seeking behaviour, a generalized linear model (GLM) with a Gaussian distribution and identity link function was fitted, treating care-seeking score as a continuous outcome. The Breusch-Pagan test indicated no evidence of heteroskedasticity (F = 0.59, *p* = 0.71), supporting the use of classical standard errors. Regression coefficients (β) were presented with 95% confidence intervals (CIs), and a *p*-value < 0.05 was considered statistically significant.

Given the low number of participants with PCR-confirmed *P. vivax* infection (n = 6), Firth logistic regression was used to reduce small-sample bias in estimating odds ratios for infection risks. Continuous covariates were dichotomised at their mean values prior to modelling. Odds ratios (OR) with corresponding 95% CIs were reported. Statistical significance of OR was determined by the exclusion of 1 from the 95% CIs.

## Results

### Sociodemographic profile of migrant participants

The mean age of participants was 34.5 years (SD: 14.5), with a slight female majority (52.7%). Most had no formal education (71.0%) and were of Karen ethnicity (71.3%). Common occupations included daily wage labour (39.0%) and agriculture (21.7%), with all participants working in the informal sector. The mean monthly household income was 4028 THB (approximately USD 122) (SD: 1971). On average, participants had been in Thailand for 89.2 days (SD: 48.4) during their current stay. Nearly half (43.6%) reported rarely returning to Myanmar. Daily wage labourers represent an occupational category, whereas daily cross-border travel refers migration frequency; these groups overlap but are not identical within the short-term migrant population studied. Most lived with family (40.3%) or alone (39.3%). A history of malaria was reported by 39.0% of participants, and none had health insurance, despite being eligible to obtain it independently, either online or at designated public hospitals. The average travel time to the nearest health facility was 19.2 min (SD: 10.8) (Table 1).

**Table 1:** Sociodemographic characteristics of study participants (n = 300)

Characteristic	Frequency (n)	Percentage (%)
Age (years)		
Mean ± SD, Min–Max	34.5 ± 14.5, 18–78	
Sex		
Female	158	52.7
Male	142	47.3
Level of education		
Illiterate/No formal	213	71.0
Primary	58	19.3
Secondary and above	29	9.7
Ethnicity		
Karen	214	71.3
Burmese	86	28.7
Occupation		
Daily wage labourer	117	39.0
Other (restaurant, hotel, etc.)	68	22.7
Agricultural worker	65	21.7
Construction worker	35	11.6
Unemployed	15	5.0
Workplace stability		
Temporary	177	59.0
Seasonal	99	33.0
Permanent	24	8.0
Type of employment		
Informal (without contract)	300	100
Estimated monthly income (THB)		
Mean ± SD, Min–Max	4028 ± 1971, 500–20000	
Days in Thailand during current visit		
Mean ± SD, Min–Max	89.2 ± 48.4, 3–168	
Frequency of return to Myanmar		
Every day	21	7
Weekly	56	18.7
Once a month	53	17.7
Once every few months	39	13
Rarely	131	43.6
Living arrangements		
Alone	118	39.3
With family	121	40.3
With friends/colleagues	61	20.4
Ever had malaria in life		
Yes	117	39.0
No	183	61.0
Health insurance coverage		
No	300	100
Distance to reach to nearest health facility (min)		
Mean ± SD, Min–Max	19.2 ± 10.8, 5–60	

1 USD ≈ 33 THB

### Distribution of malaria knowledge, attitudes, and care-seeking scores

Detailed item-level descriptive statistics for malaria knowledge (Supplementary Table S1), attitudes (Supplementary Fig. S1), and care-seeking practices (Table 2) are presented. Briefly, over 60% of participants reported importance of seeking medical care immediately when experiencing fever or chills; however, only 35% sought care within 24 h of suspected malaria symptoms. Public health facilities were the preferred source of treatment (98.0%), and only half of the participants (50.3%) reported ever visiting a health facility for suspected malaria symptoms (Table 2).

**Table 2:** Malaria care-seeking practices among Myanmar migrants (n = 300)

Malaria care-seeking question	n (%)
When you experience fever or chills, do you seek medical care?	
Yes, immediately	191 (63.7)
Only if symptoms worsen	72 (24.0)
No, I treat it at home	37 (12.3)
Have you ever visited a health facility for suspected malaria symptoms?	
Yes	151 (50.3)
No (skip to next question.)	149 (49.7)
Did you follow the prescribed treatment course if diagnosed with malaria? (n = 151)	
Yes	119 (78.8)
Partially	18 (18.5)
No	4 (2.6)
Where would you go for treatment if you think you have malaria?	
Public health facility	294 (98.0)
Private clinic	4 (1.3)
Pharmacy	29 (9.7)
Traditional healer	6 (2.0)
Self-medication	12 (4.3)
How long do you typically wait before seeking medical help when you suspect malaria?	
Within 24 h	105 (35.0)
2–3 days	156 (52.0)
A week or more	39 (13.0)
Do you take any preventive measures to avoid malaria?	
Bed nets	154 (51.3)
Insecticide-treated nets	126 (42.0)
Avoid drinking contaminated water	13 (4.3)
Mosquito repellents	81 (27.0)
Wearing long-sleeve clothing	96 (32.0)
Burning mosquito coils	54 (18.0)
Not at all	11 (3.7)
Have you ever bought anti-malaria medication from a pharmacy without a doctor’s prescription?	
Yes	44 (14.7)
No	256 (85.3)
Do you know of any community health workers or volunteers who assist with malaria prevention or treatment in your area?	
Yes	186 (62.0)
No	114 (38.0)
How confident are you in the quality of care provided by healthcare facilities in treating malaria?	
Very confident	249 (83.0)
Somewhat confident	37 (12.3)
Not confident	4 (1.3)
I don’t know	10 (3.3)
Have you ever faced challenges in accessing healthcare for malaria treatment (e.g., transportation, cost, lack of time)?	
Yes	57 (19.0)
No	243 (81.0)

For questions allowing multiple responses, totals may exceed 100% because of multiple selections per participant

**Fig. 2:**
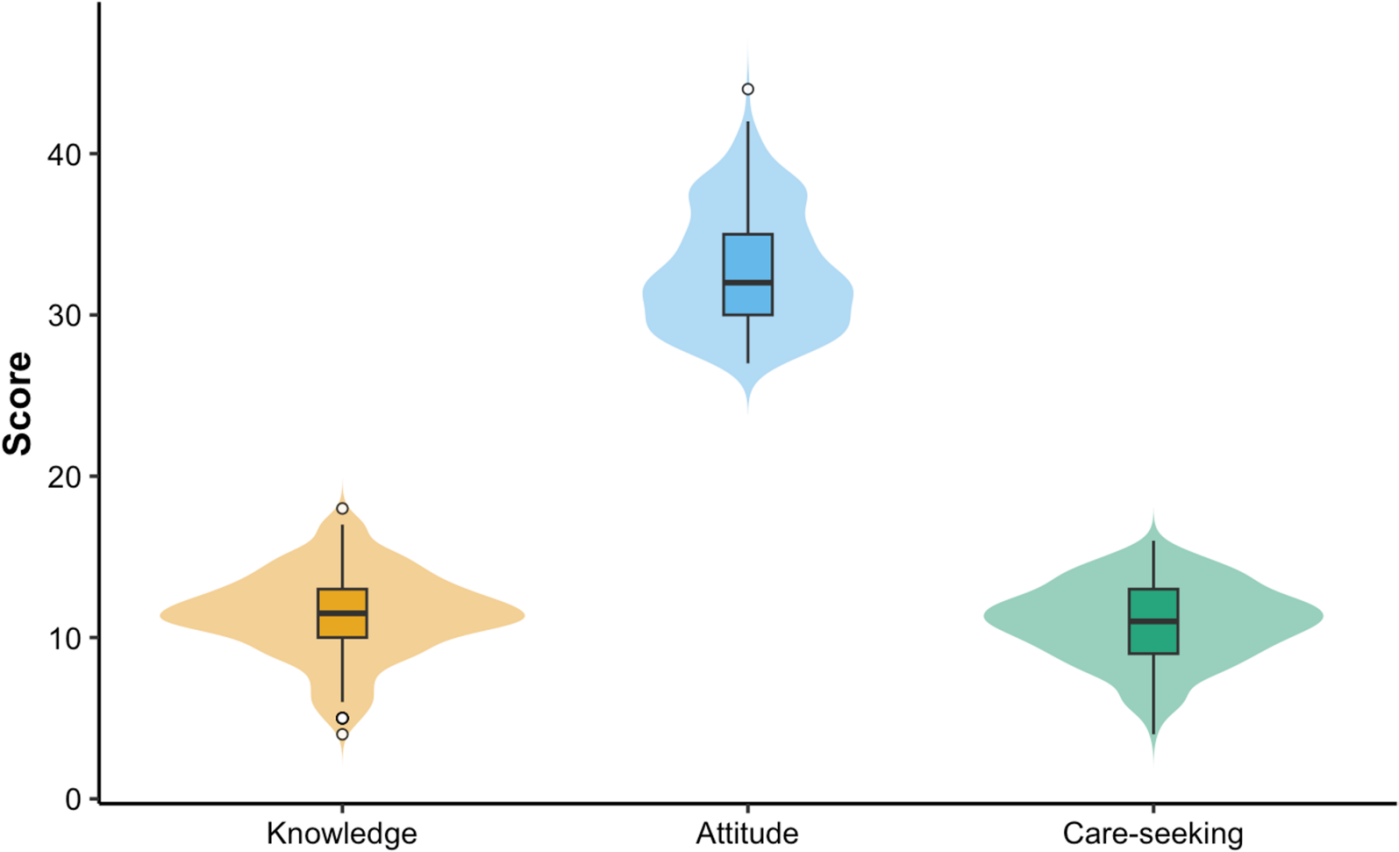
Distribution of malaria-related knowledge, attitudes, and care-seeking scores among short-term Myanmar migrants in western Thailand. Violin plots display the full distribution of scores, overlaid with boxplots representing the interquartile range (IQR), where the horizontal line indicates the median. Whiskers extend to 1.5 times the IQR, and dots represent outliers. Higher scores indicate greater malaria knowledge, more favourable attitudes, and stronger care-seeking intentions. Knowledge scores showed greater variability and more low-end outliers, while attitudes were more consistently clustered around the median. Scores across the three domains are not directly comparable because each domain was measured on a different scale

Figure 2 shows the distribution of scores related to malaria knowledge, attitudes, and care-seeking practices. Scores were compared internally within the migrant population studied because no non-migrant comparator group was included. The mean knowledge score was 11.4 (SD: 2.64; range: 4–18) out of a possible 24 points, indicating generally low knowledge levels. Attitude scores were higher, with a mean of 32.8 (SD: 3.64; range: 27–44) out of 44 possible points, reflecting generally favourable attitudes toward malaria prevention and control. Care-seeking scores had a mean of 11.0 (SD: 2.37; range: 4–16) out of a possible 18, suggesting moderate levels of appropriate care-seeking behaviour.

### Factors associated with malaria care-seeking behaviour

Among Myanmar migrants, higher malaria care-seeking scores were significantly associated with being a daily wage labourer (β = 0.66, 95% CI 0.01–1.31, *p* = 0.049), having had malaria in the past (β = 1.08, 95% CI 0.59–1.58, *p* < 0.001), and higher malaria knowledge scores (β = 0.34, 95% CI 0.24–0.44, *p* < 0.001). In contrast, Karen ethnicity was negatively associated with care-seeking behaviour compared to Burmese ethnicity (β = –0.95, 95% CI − 1.74 to − 0.16, *p* = 0.018). Additionally, migrants who reported returning to Myanmar only once every few months had significantly higher care-seeking scores than those crossing daily (β = 1.34, 95% CI 0.05–2.62, *p* = 0.042). No significant associations were observed for age, sex, education level, income, distance to health facility, or attitude scores (Table 3).

**Table 3:** Factors associated with malaria care-seeking among Myanmar migrants (n = 300)

Characteristic	Estimate (β)	Std. Error	95% CI	*p*-value
Age (years)	0.00	0.01	− 0.02, 0.02	0.783
Sex				
Female	Ref.			
Male	0.51	0.27	− 0.02, 1.03	0.062
Education level				
Illiterate/No formal	Ref.			
Primary	0.64	0.33	− 0.01, 1.29	0.055
Secondary and above	0.59	0.45	− 0.29, 1.48	0.189
Ethnicity				
Burmese	Ref.			
Karen	− 0.95	0.40	1.74, − 0.16	0.018*
Occupation				
Agricultural worker	Ref.			
Construction worker	− 0.22	0.43	− 1.07, 0.62	0.603
Daily wage labourer	0.66	0.33	0.01, 1.31	0.049*
Other (restaurant, hotel, etc.)	0.59	0.40	− 0.20, 1.38	0.145
Unemployed	− 0.89	0.64	− 2.15, 0.37	0.167
Workplace stability				
Permanent	Ref.			
Seasonal	− 0.32	0.53	− 1.37, 0.73	0.547
Temporary	0.19	0.51	− 0.81, 1.19	0.710
Monthly income (THB)	0.00	0.00	0.00, 0.00	0.681
Days in Thailand	0.00	0.00	− 0.01, 0.01	0.780
Frequency of return to Myanmar				
Every day	Ref.			
Weekly	0.34	0.57	− 0.78, 1.47	0.548
Once a month	0.58	0.61	− 0.62, 1.78	0.343
Once every few months	1.34	0.65	0.05, 2.62	0.042*
Rarely	0.74	0.61	− 0.45, 1.94	0.225
Living arrangements				
Alone	Ref.			
With family	0.37	0.28	− 0.17, 0.92	0.183
With friends/colleagues	− 0.10	0.32	− 0.72, 0.52	0.757
Ever had malaria in life				
No	Ref.			
Yes	1.08	0.25	0.59, 1.58	< 0.001*
Distance to health facility (min)	0.01	0.01	− 0.02, 0.04	0.391
Knowledge score	0.34	0.05	0.24, 0.44	< 0.001*
Attitude score	0.05	0.04	− 0.03, 0.13	0.233

1 USD ≈ 33 THB; *Significance at *p* < 0.05 by generalized linear model

### Factors associated with malaria infection

Among 300 participants, the prevalence of malaria was low, with only six (2%) *P. vivax* cases detected. All six individuals were afebrile at the time of testing and reported no recent malaria-related symptoms. None had sought healthcare prior to study screening. The majority were of Karen ethnicity (5/6), worked as daily wage labourers (4/6), and had little or no formal education (4/6). Most lived with family (4/6) and returned to Myanmar frequently (4/6).

Crude odds ratios were estimated using Firth logistic regression to account for small event counts. Participants with lower malaria knowledge scores (< 11.4) had significantly higher odds of infection compared to those with higher knowledge (OR = 13.5; 95% CI 1.58–177). Similarly, participants with lower care-seeking scores (< 11.0) were more likely to be infected (OR = 5.86; 95% CI 1.15–57.7). No other sociodemographic or behavioural factors were significantly associated with infection (Table 4).

**Table 4:** Factors associated with malaria infection among Myanmar migrants (n = 300)

Characteristic	Malaria infection by PCR		OR (95% CI)
	Negative (n = 294)	Positive (n = 6)	
	n (%)	n (%)	
Age (years)			
< 35	171 (98.3)	3 (1.7)	Ref.
≥ 35	123 (97.6)	3 (2.4)	1.39 (0.29–6.65)
Sex			
Female	155 (98.1)	3 (1.9)	Ref.
Male	139 (97.9)	3 (2.1)	1.11 (0.23–5.33)
Education			
Illiterate/No formal	209 (98.1)	4 (1.9)	Ref.
Primary and above	85 (97.7)	2 (2.3)	1.36 (0.23–6.26)
Ethnicity			
Burmese	85 (98.8)	1 (1.2)	Ref.
Karen	209 (97.7)	5 (2.3)	1.50 (0.29–14.7)
Occupation			
Daily wage labourer	113 (96.6)	4 (3.4)	Ref.
Other (Agricultural, restaurant, hotel, etc.)	181 (98.9)	2 (1.1)	0.35 (0.06–1.59)
Workplace stability			
Permanent	120 (97.6)	3 (2.4)	Ref.
Temporary/Seasonal	174 (98.3)	3 (1.7)	0.69 (0.14–3.31)
Monthly income (THB)			
≥ 4028	85 (98.8)	1 (1.2)	Ref.
< 4028	209 (97.7)	5 (2.3)	1.50 (0.29–14.7)
Days in Thailand			
≥ 89.2	164 (97.0)	5 (3.0)	Ref.
< 89.2	130 (99.2)	1 (0.8)	0.34 (0.03–1.75)
Return to Myanmar			
At least monthly	126 (96.9)	4 (3.1)	Ref.
Rarely	168 (98.8)	2 (1.2)	0.42 (0.07–1.91)
Living arrangements			
Alone	116 (98.3)	2 (1.7)	Ref.
With family/friends	178 (97.8)	4 (2.2)	1.17 (0.26–6.83)
Malaria history			
No	178 (97.3)	5 (2.7)	Ref.
Yes	116 (99.2)	1 (0.9)	0.42 (0.04–2.13)
Distance to health facility (min)			
≥ 19.2	102 (99.0)	1 (1.0)	Ref.
< 19.2	192 (97.5)	5 (2.5)	1.95 (0.38–19.2)
Knowledge score			
≥ 11.4	150 (100)	0 (0.0)	Ref.
< 11.4	144 (96.0)	6 (4.0)	13.5 (1.58–177)
Attitude score			
≥ 32.8	132 (97.8)	3 (2.2)	Ref.
< 32.8	162 (98.2)	3 (1.8)	0.82 (0.17–3.90)
Care-seeking score			
≥ 11.0	181 (99.5)	1 (0.6)	Ref.
< 11.0	113 (95.8)	5 (4.2)	5.86 (1.15–57.7)

1 USD ≈ 33 THB; OR: Odds ratio; 95% CI 95% Confidence interval

## Discussion

This study identified sociodemographic factors, migration patterns, and malaria knowledge as key determinants of malaria care-seeking behaviours among short-term Myanmar migrants in Thailand. Karen individuals were less likely to seek care than other groups [15, 18]. Their longer-term residence near the border may provide greater familiarity with the local language and healthcare system [15], which paradoxically may reduce perceived urgency to seek treatment for mild symptoms [40]. According to the Health Belief Model [41], individuals living in areas with predominantly *P. vivax* transmission may perceive malaria as less severe, lowering motivation to seek prompt care. Migrants from border states enter Thailand through informal routes in search of survival livelihoods. Compared with ethnic groups arriving from central or non-border areas, especially recent arrivals, long-term border residents may view malaria as a manageable issue, knowing where and how to access diagnosis and treatment.

Economic drivers also shaped care-seeking behaviour. Although income level was not statistically associated with care-seeking scores, daily wage labourers reported higher scores, possibly due to concerns about income loss from illness-related absences [18]. Without health insurance or legal documentation, many migrants lack access to larger public hospitals in Thailand [42]. Although malaria services are provided free of charge at field-based clinics and posts, migrants often face out-of-pocket costs when seeking care at other public or private facilities. The need to maintain stable employment may motivate daily wage workers to seek care promptly, even in the absence of symptoms.

Migrants who returned to Myanmar only once every few months had higher care-seeking scores than those travelling more frequently. This may reflect increased exposure to malaria elimination efforts and health messaging in Thailand, such as the 1-3-7 surveillance strategy implemented since 2016, rather than participation in the one-time active case detection screening conducted for this study, as care-seeking scores were based solely on self-reported practices for managing fever or suspected malaria [43]. In contrast, malaria services in conflict-affected areas of Myanmar often rely on non-governmental organisations and may be limited, inconsistent, and may only partially reach affected populations [23, 44]. Migrants residing longer in Thailand may have more contact with community health workers and field-based interventions, resulting in greater awareness and engagement with malaria services. Given that infection risk is lower in Thailand, their lower perceived risk may be appropriate and reflective of the local transmission context.

Individuals with a history of malaria infection demonstrated stronger care-seeking behaviours, likely due to increased confidence in the effectiveness of treatment [45]. First-hand experience with illness may enhance recognition of symptoms and the urgency of timely treatment. Health education strategies that leverage peer sharing and patient networks could help promote this knowledge more broadly within communities. Materials and tools should be developed in migrants’ native languages to improve understanding and cultural relevance [15, 44].

Consistent with previous research, malaria knowledge was closely linked to care-seeking behaviour [46–49]. One study further supports this finding, emphasising the interconnected nature of awareness, attitudes, and behaviours [50]. Individuals with greater malaria knowledge are more likely to seek care appropriately. As migrants may not always access conventional malaria control services, targeted health promotion initiatives are needed [44]. Multilingual mobile applications and other contactless interventions may be especially useful in this context. Increasing the number of malaria service posts near informal border-crossing points may also improve access and reduce transmission in these high-risk areas.

Participants with limited knowledge and low care-seeking scores were more likely to have asymptomatic *P. vivax* infections. Individuals residing in endemic regions for extended periods may develop partial immunity from repeated infections, resulting in subclinical presentations [8]. Limited knowledge and inappropriate care-seeking behaviours may also lead to reliance on self-medication or unregulated sources, raising concerns about inadequate treatment [15, 23–27]. Such asymptomatic infections are common in partially immune populations and often reflect subclinical carriage rather than poor health-seeking behaviour, as individuals without symptoms typically have no reason to seek care. Asymptomatic carriers can still contribute to transmission, [51, 52] highlighting the need for targeted surveillance, preventive interventions, and timely treatment among mobile populations. The small number of infections in this study limited the feasibility of multivariate modelling, and results should therefore be interpreted with caution. Future studies incorporating seroprevalence data may be valuable, as serological markers provide insights into historical exposure and identify areas of persistent transmission often missed by routine surveillance [53]. The emerging serology-guided targeted treatment (SeroTAT) approach, which leverages serological data to target interventions, could also strengthen malaria elimination strategies [54].

To the best of available knowledge, this is the first study to assess malaria care-seeking scores and infection prevalence specifically among short-term Myanmar migrants, a group often excluded from routine surveillance and control efforts. As a cross-sectional study based on self-reported questionnaire data, findings may not be generalisable to all migrant populations. Seasonal mobility, environmental risks, and malaria endemicity in migrants’ areas of origin may also influence results. Documentation status was not assessed due to ethical constraints, although undocumented migrants may face greater barriers to care. Other barriers, including perceived discrimination, fear of treatment side effects, and transportation challenges, have been reported previously [45, 55, 56] and merit further investigation. Future research should also include children, whose exposure patterns and care-seeking behaviours may differ from adults and have implications for elimination strategies. Qualitative research could further explore reasons for delayed or insufficient care-seeking in this vulnerable population.

## Conclusions

Despite high awareness of malaria symptoms and generally positive attitudes toward malaria, timely care-seeking among short-term Myanmar migrants in Thailand remains suboptimal, with limited utilisation of health services. Health-seeking behaviours were significantly influenced by socioeconomic status, migration patterns, and malaria knowledge. Although malaria prevalence was low, the detection of asymptomatic *P. vivax* infections highlights the potential for ongoing transmission. Targeted surveillance and strengthened community-based health education, particularly among marginalised ethnic groups, are essential to improve care-seeking behaviours and support malaria elimination efforts in border regions.

## Supplementary Information

Additional file 1.

Additional file 2.

## Data Availability

All data generated or analysed during this study are included in the article. The de-identified raw dataset is available from the corresponding author upon reasonable request.
