# Supplementary material for: Malaria care-seeking behaviours and infection prevalence among short-term Myanmar migrants in Thailand
Source: Malar J. 2025 Sep 1;24:280. doi: 10.1186/s12936-025-05539-8 (PMC12400559; doi:10.1186/s12936-025-05539-8)
Supplement: Supplementary file 1 — Additional file 1. [file 12936_2025_5539_MOESM1_ESM.docx]

**Questionnaire for malaria care-seeking practices among Myanmar migrants**

Participant ID: ___________________

Date: __________________

**Part 1. Socioeconomic characteristics**

| **#** | **Characteristics** | **Answers** |
| --- | --- | --- |
| 1 | How old are you? | _______________years |
| 2 | Gender | - Male  - Female |
| 3 | Level of education | - Illiterate/No formal education  - Primary school  - Secondary school  - High school  - College and above |
| 4 | Ethnicity | - Burmese  - Karen  - Mon  - Other (please specify)  _____________________________ |
| 5 | Citizenship | - Thai  - Myanmar  - Other (please specify)  ______________________________ |
| 6 | Occupation | - Daily wage laborer  - Agricultural worker  - Construction worker  - Service industry (restaurant, hotel, etc.)  - Unemployed  - Other (please specify)  _____________________________ |
| 7 | Workplace stability | - Temporary  - Permanent  - Seasonal |
| 8 | Type of employment | - Formal (with contract)  - Informal (without contract) |
| 9 | Estimated monthly family income | _________________THB |
| 10 | How many days have you been in Thailand during your current visit? | _________________days |
| 11 | Frequency of return to Myanmar | - Every day  - 4 to 6 days per week  - 1 to 3 days per week  - Once a week  - 2 to 3 times per month  - Once a month  - Once every few months  - Rarely |
| 12 | Living arrangements | - Alone  - With family  - With friends/colleagues |
| 13 | Have you ever had malaria in your life? | - Yes  - No |
| 14 | Health insurance coverage | - Yes  - No |
| 15 | Distance to reach to nearest health facility | _____________________minutes |

**Part 2. Knowledge about malaria-care seeking**

| **#** | **Questions** | **Answers** |
| --- | --- | --- |
| 1 | What are the common symptoms of malaria? (select all that apply) | - Fever  - Chills  - Headache  - Body aches  - Coughing/Sneezing  - Other (please specify)  _________________________________ |
| 2 | **How is malaria transmitted?** (select all that apply) | - By mosquito bites  - Through contaminated water  - By contact with an infected person  - Eating fruits such as papayas/bananas  - Other (please specify)  __________________________________ |
| 3 | How can we diagnose malaria? (select all that apply) | - Through a blood test  - By visiting a healthcare facility  - By visiting a hospital  - It cannot be diagnosed  - Based on previous experience  - Based on symptoms  - Other (please specify)  _____________________________________ |
| 4 | How soon after recognizing malaria symptoms should a person seek treatment? | - Immediately  - 1-2 days later  - After a week  - Only if symptoms become severe  - Other (please specify)  _____________________________________ |
| 5 | Do you think untreated malaria can be spread to others? | - Yes  - No |
| 6 | Do you believe that early treatment can prevent serious complications of malaria? | - Yes  - No |
| 7 | **Do you know of any healthcare services in your area where people can receive malaria treatment, either free or paid?** | - Yes  - No |
| 8 | Do you trust healthcare providers to give accurate diagnosis and treatment for malaria? | - Yes  - No |
| 9 | Which health facility is the most appropriate place to seek treatment for malaria? | - Government hospital  - Private clinic  - Pharmacy  - Traditional healer  - Village health volunteers  - Malaria clinics |
| 10 | What should you do if the malaria symtpoms are not relieved after a few days? (select all that apply) | - Visit a healthcare provider again  - Use other medications at home  - Seek traditional remedies  - Do nothing and wait for symptoms to go away |
| 11 | What do you think are the consequences of delaying or not seeking proper care for malaria? (select all that apply) | **- Severe illness or complications**  - Death  - Increased risk of spreading malaria to others  - Prolonged symptoms and suffering  - Higher medical costs later  - None, malaria will go away on its own  - I don’t know  - Other (please specify)  ___________________________________ |

**Part 3. Perceptions toward malaria care-seeking**

| **#** | **Statements** | **Strongly disagree** | **Disagree** | **Agree** | **Strongly agree** |
| --- | --- | --- | --- | --- | --- |
|  |  | 1 | 2 | 3 | 4 |
| 1 | I will receive effective treatment if I go to a health facility for malaria. |  |  |  |  |
| 2 | It is important to seek medical care as soon as malaria symptoms appear. |  |  |  |  |
| 3 | Healthcare providers can accurately diagnose malaria. |  |  |  |  |
| 4 | Going to a healthcare facility could result in getting caught by the police.* |  |  |  |  |
| 5 | **Seeking care at a health facility is the best way to prevent serious malaria complications.** |  |  |  |  |
| 6 | There is no need to visit a health facility for malaria unless the symptoms are very severe.* |  |  |  |  |
| 7 | I believe malaria can be treated at home without visiting a health facility.* |  |  |  |  |
| 8 | Going to a health facility for malaria care is too expensive for me.* |  |  |  |  |
| 9 | I prefer using traditional remedies for malaria instead of going to a health facility.* |  |  |  |  |
| 10 | I feel comfortable discussing my symptoms with healthcare workers when I suspect malaria. |  |  |  |  |
| 11 | Malaria can spread to others thorugh mosquito bites if left untreated? |  |  |  |  |

*Negative statements.

**Part 4. Care-seeking practices regarding malaria**

| **#** | **Questions** | **Answers** |
| --- | --- | --- |
| 1 | When you experience fever or chills, do you seek medical care? | - Yes, immediately  - Only if symptoms worsen  - No, I treat it at home |
| 2a | Have you ever visited a health facility for suspected malaria symptoms? | - Yes  - No (skip to Q3.) |
| 2b | Did you follow the prescribed treatment course if diagnosed with malaria? | - Yes  - Partially  - No |
| 3 | Where would you go for treatment if you think you have malaria? (select all that apply) | - Public health facility  - Private clinic  - Pharmacy  - Traditional healer  - Self-medication  - Other (please specify)  _____________________________ |
| 4 | How long do you typically wait before seeking medical help when you suspect malaria? | - Within 24 hours  - 2–3 days  - A week or more |
| 5 | Do you take any preventive measures to avoid malaria? (select all that apply) | - Bed nets  - Insecticide-treated nets  - Avoid drinking contaminated water  - Mosquito repellents  - Wearing long-sleeve clothing  - Burning mosquito coils  - Not at all  - Other (please specify)  ______________________________ |
| 6 | Have you ever bought anti-malaria medication from a pharmacy without a doctor’s prescription? | - Yes  - No |
| 7 | Do you know of any community health workers or volunteers who assist with malaria prevention or treatment in your area? | - Yes  - No |
| 8 | How confident are you in the quality of care provided by healthcare facilities in treating malaria? | - Very confident  - Somewhat confident  - Not confident  - I don’t know |
| 9 | Have you ever faced challenges in accessing healthcare for malaria treatment (e.g., transportation, cost, lack of time)? | - Yes  - No |
