# Supplementary material for: Malaria care-seeking behaviours and infection prevalence among short-term Myanmar migrants in Thailand
Source: Malar J. 2025 Sep 1;24:280. doi: 10.1186/s12936-025-05539-8 (PMC12400559; doi:10.1186/s12936-025-05539-8)

**Supplementary table 1** Malaria knowledge among Myanmar migrants (n=300)

| **Knowledge question** | **n (%)** |
| --- | --- |
| **What are the common symptoms of malaria?** |  |
| Fever | 288 (96.0) |
| Chills | 175 (58.3) |
| Headache | 264 (88.0) |
| Body aches | 141 (47.0) |
| Coughing/Sneezing | 14 (4.7) |
| **How is malaria transmitted?** |  |
| By mosquito bites | 278 (92.7) |
| Through contaminated water | 41 (13.7) |
| By contact with an infected person | 49 (16.3) |
| Eating fruits such as papayas/bananas | 12 (4.0) |
| **How can we diagnose malaria?** |  |
| Through a blood test | 127 (42.3) |
| By visiting a healthcare facility | 139 (46.3) |
| By visiting a hospital | 138 (46.0) |
| It cannot be diagnosed | 1 (0.3) |
| Based on previous experience | 49 (16.3) |
| Based on symptoms | 69 (23.0) |
| **How soon after recognizing malaria symptoms should a person seek treatment?** | |
| Immediately | 82 (27.3) |
| 1-2 days later | 151 (50.3) |
| After a week | 32 (10.7) |
| Only if symptoms become severe | 24 (8.0) |
| **Do you think untreated malaria can be spread to others?** |  |
| Yes | 230 (76.7) |
| No | 70 (23.3) |
| **Do you believe that early treatment can prevent serious complications of malaria?** | |
| Yes | 266 (88.7) |
| No | 34 (11.3) |
| **Do you know of any healthcare services in your area where people can receive malaria treatment, either free or paid?** | |
| Yes | 233 (77.7) |
| No | 67 (22.3) |
| **Do you trust healthcare providers to give accurate diagnosis and treatment for malaria?** | |
| Yes | 294 (98.0) |
| No | 6 (2.0) |
| **Which health facility is the most appropriate place to seek treatment for malaria?** | |
| Government hospital | 129 (43.0) |
| Pharmacy | 17 (5.7) |
| Traditional healer | 5 (1.7) |
| Village health volunteers | 43 (14.3) |
| Malaria clinics | 148 (49.3) |
| **What should you do if the malaria symptoms are not relieved after a few days?** (select all that apply) | |
| Visit a healthcare provider again | 150 (50%) |
| Use other medications at home | 169 (56.3) |
| Seek traditional remedies | 34 (11.3) |
| Do nothing and wait for symptoms to go away | 59 (19.7) |
| **What do you think are the consequences of delaying or not seeking proper care for malaria?** | |
| **Severe illness or complications** | 115 (38.3) |
| Death | 122 (40.7) |
| Increased risk of spreading malaria to others | 37 (12.3) |
| Prolonged symptoms and suffering | 45 (15.0) |
| Higher medical costs later | 18 (6.0) |
| None, malaria will go away on its own | 3 (1.0) |
| I don’t know | 80 (26.7) |

For questions allowing multiple responses, totals may exceed 100% because of multiple selections per participant.

**Supplementary Fig. 1** Malaria attitudes among Myanmar migrants (n=300). The figure displays a horizontal stacked bar chart summarizing responses to 11 attitude statements related to malaria care and beliefs. Each bar represents the proportion of participants who strongly disagreed, disagreed, agreed, or strongly agreed with each statement. Statements marked with an asterisk indicate negative attitudes.


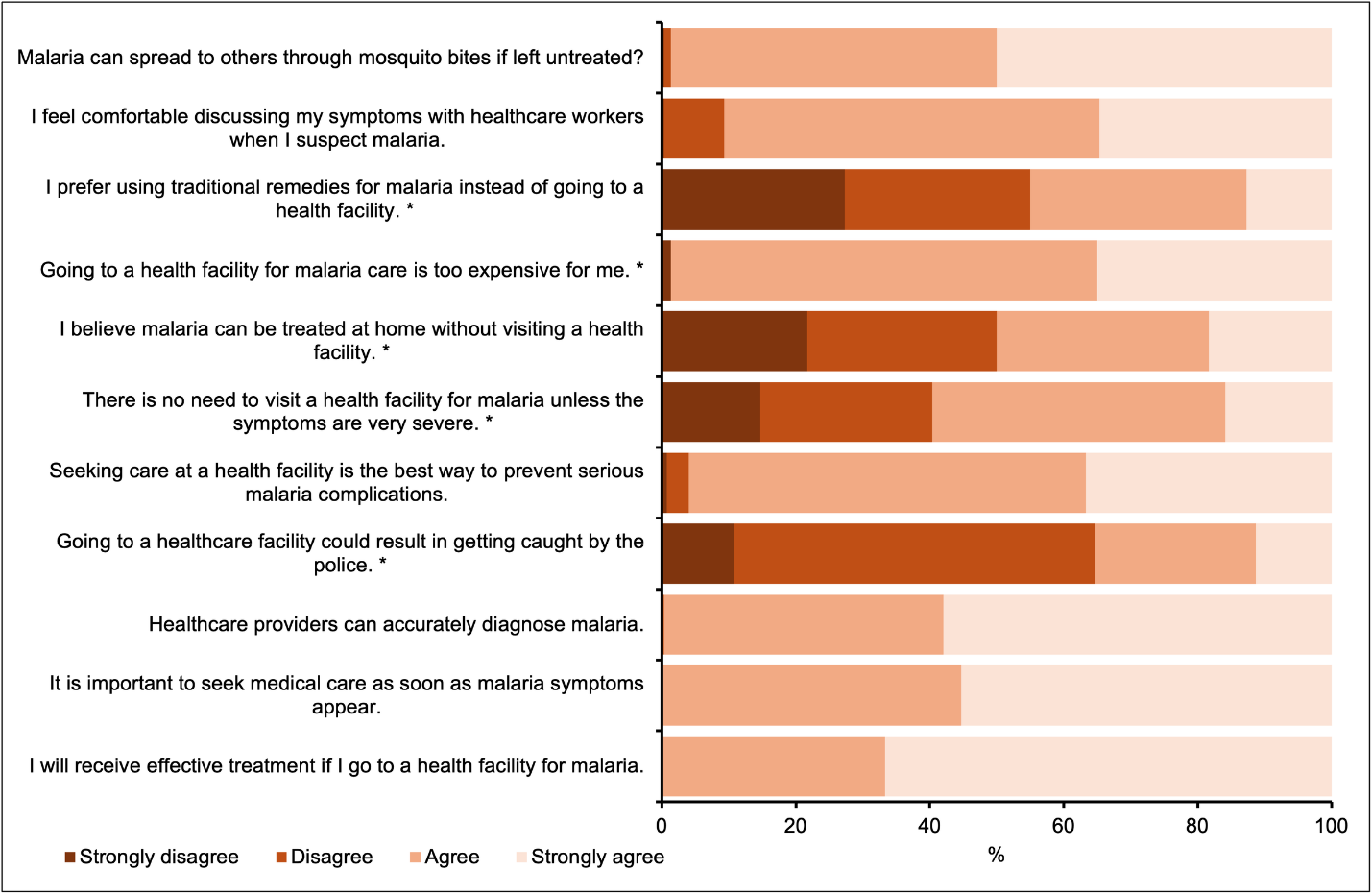

Supplement: Supplementary file 2 — Additional file 2. [file 12936_2025_5539_MOESM2_ESM.docx]
